# Ramanomics: New Omics Disciplines Using Micro Raman Spectrometry with Biomolecular Component Analysis for Molecular Profiling of Biological Structures

**DOI:** 10.3390/bios7040052

**Published:** 2017-11-15

**Authors:** Andrey N. Kuzmin, Artem Pliss, Paras N. Prasad

**Affiliations:** 1 Institute for Lasers, Photonics and Biophotonics, University at Buffalo, State University of New York, Buffalo, NY 14260-3000, USA; ampliss@buffalo.edu; 2 Advanced Cytometry Instrumentation Systems, LLC, 640 Ellicott Street—Suite 499, Buffalo, NY 14203, USA

**Keywords:** Raman spectrometry, microscopy, biomolecular analysis, single cell assay

## Abstract

Modern instrumentation for Raman microspectroscopy and current techniques in analysis of spectral data provide new opportunities to study molecular interactions and dynamics at subcellular levels in biological systems. Implementation of biomolecular component analysis (BCA) to microRaman spectrometry provides basis for the emergence of Ramanomics, a new biosensing discipline with unprecedented capabilities to measure concentrations of distinct biomolecular groups in live cells and organelles. Here we review the combined use of microRaman-BCA techniques to probe absolute concentrations of proteins, DNA, RNA and lipids in single organelles of live cells. Assessing biomolecular concentration profiles of organelles at the single cell level provides a physiologically relevant set of biomarkers for cellular heterogeneity. In addition, changes to an organelle’s biomolecular concentration profile during a cellular transformation, whether natural, drug induced or disease manifested, can provide molecular insight into the nature of the cellular process.

## 1. Introduction

Molecular organization and regulation of eukaryotic cells is based on highly organized subcellular structures of specialized functions i.e., organelles. To advance the understanding of cellular regulation and disease development, there is a high demand for bioanalytical techniques capable of studying the link between the subcellular biomolecular composition of organelles and physiologic or pathologic cell functions. Currently, this demand is met by proteomics, metabolomics, lipidomics and other “omics” disciplines [1,2], where quantitative profiling of subcellular compartments is performed mostly by mass spectrometry. Using this technique, several thousand diverse molecular species were identified in various cellular compartments. However, a major shortcoming of mass-spectroscopy is insufficient sensitivity for analysis of single organelles. Omics studies are reliable when the sample mass exceeds a certain amount, usually in the nanogram range; thus it involves the collection of organelles from a large number of cells. This approach masks biochemical variations between individual organelles and requires subcellular fractionation, separation, and purification of organelles; therefore, it is not compatible with live systems. Besides, organelles are not stationary but rather dynamic organizations interacting with each other that change with time, which requires, again, noninvasive approaches for biomolecular probing in situ.

Raman light scattering from different types of biomolecules generates corresponding bands in the Raman spectra, which enables to resolve all the diversity of biomolecular components—nucleic acids, amino acids, proteins, and various classes of lipids and saccharides. Being applied to live cells, it provides multiple benefits for molecular analysis, including the linearity of this optical phenomenon, which allows for quantitative probing of biomolecular distribution [3,4]. This makes confocal Raman spectrometry, apparently, the only in situ technique, capable of providing quantitative biochemical analysis of a single live cell.

Technological advances in Raman spectroscopy hardware (e.g., lasers, solid-state photodetectors, etc.) have significantly boosted the sensitivity of this analytical method. Since the time when the first studies on living cells were performed using Raman microspectroscopy [5], Raman scattering has found countless applications in biomedical research and clinical diagnosis. The capabilities and achievements of confocal Raman technique in the diversified biomedical sector as well as its barriers and limitations have extensively been reviewed [6,7,8,9,10,11,12,13,14,15,16,17,18,19,20,21,22,23]. These achievements create a potential for emergence of Ramanomics as an independent omics discipline. Similar to other omics approaches, Ramanomics requires specific strategies and protocols for sample analysis. The most important procedure for biomolecular analysis includes the methods of deconvolution of Raman spectrum to its spectral constituents. In fact, spectral deconvolution for quantitative purposes has been used for several years in the pharmaceutical industry [24,25].

For bioapplications this method is known as Biomolecular Component Analysis (BCA), a powerful algorithm that identifies the concentration of different molecular groups collectively contributing to the Raman spectrum of the biological sample. BCA is successfully used in a wide range of studies, including biomolecular probing of single cells and tissue samples. Being applied to different cellular organelles, BCA creates a solid foundation for subcellular Ramanomics. Although it is well known, the novelty of BCA, highlighted in this review, is the scoring the spectral features of the organelles in order to understand and identify the cellular states and different cell types. Biomolecules (e.g., lipids, protein, etc.) and their ratios present in different organelles give rise to a library of spectral patterns, which may be used to define unique cell types or cellular states. Recent progress in BCA development and further perspectives of this technique are discussed below.

## 2. Place of BCA among the Methods of Raman Modeling

The Raman spectrum of a live cell carries unique information about the molecular composition of volume, where light interacts with cellular constituents. This information can be extracted by using a set of mathematical techniques, which may be incorporated in modern spectrometers as statistical toolboxes, or as stand-alone analytical software. These techniques, or multivariate analysis tools are discussed in details in several original papers and reviews on Raman spectroscopy and could be found elsewhere [22]. Briefly, a multivariate analysis is based on Raman spectrum modeling, which, in turn, involves linear analysis. This modeling relies on the assumptions, that the Raman spectrum of a mixture of biomolecules is a linear superposition of the mixture’s component spectra, and there is a linear dependence between the Raman signal intensity and the corresponding component concentration. There are several types of explicit (Ordinary Least-Squares) [26], implicit (Principal Component Regression, Principal Component Analysis) [27] and hybrid (Hybrid Linear Analysis) [28] methods. A major difference between these approaches is that when using explicit methods, prior knowledge of all resolvable chemical components of the mixture is required. In contrast, implicit methods do not require prior knowledge about full set of components. Thus, explicit techniques are best to use if the spectral components of the system are completely characterized, while implicit techniques are suitable when information about some spectral component is missing. Implicit methods for analysis of Raman data in biomedical applications with simplified requirements, where the knowledge about the full set of chemical components is not available, make them more commonly used.

One of the most popular implicit methods, Principal Component Analysis (PCA), creates a small set of specially chosen base spectra, i.e., the principal components, which explain/reveal all of the spectral changes in a sample’s Raman spectrum. The first principal component carries the maximum variance in the data, while the last principal component represents mostly noise. A linear combination of the weighted principal components serves as a model of the measured spectra. If spectral bands of the principal component overlap with that of specific biomolecular spectrum, then the principal component weights (scores) can be used for evaluation of relative concentration of such chemical. The results from PCA are commonly used in various cluster analyses of spectral variations. For some medical conditions, when the changes in the Raman spectra correlate with the disease progression, this cluster analysis accurately provides a valuable database to classify samples, clinical diagnostics and staging [6,22]. However, due to the way in which the principal components are generated, they, as mathematical objects, do not represent real molecular spectra, and, therefore, PCA neither provides precise information about the biochemical variations that caused the spectral changes, nor a quantitative molecular composition of the studied sample. In contrast, the explicit BCA, is based on an accurate spectral fit of the measured Raman spectrum to a model spectrum, generated by a linear summation of the weighted spectra of the basic molecular components [29,30,31,32,33,34,35,36,37,38,39,40]. In mathematical terms, the Raman spectrum of a measured cellular domain $r_{total}=c_{1}r_{1}+c_{2}r_{2}+\ldots +c_{i}r_{i}$, where $r_{i}$ is the Raman spectrum and $c_{i}$ is a weight of the *i*th component. The weights, or the specific contributions of the each component into model $r_{total}$ relate to the macromolecular concentrations. If all component spectral profiles, $r_{i}$, are known, the model can then be generated by any mathematical fitting, such as the least-squares algorithm.

**Figure 1:**
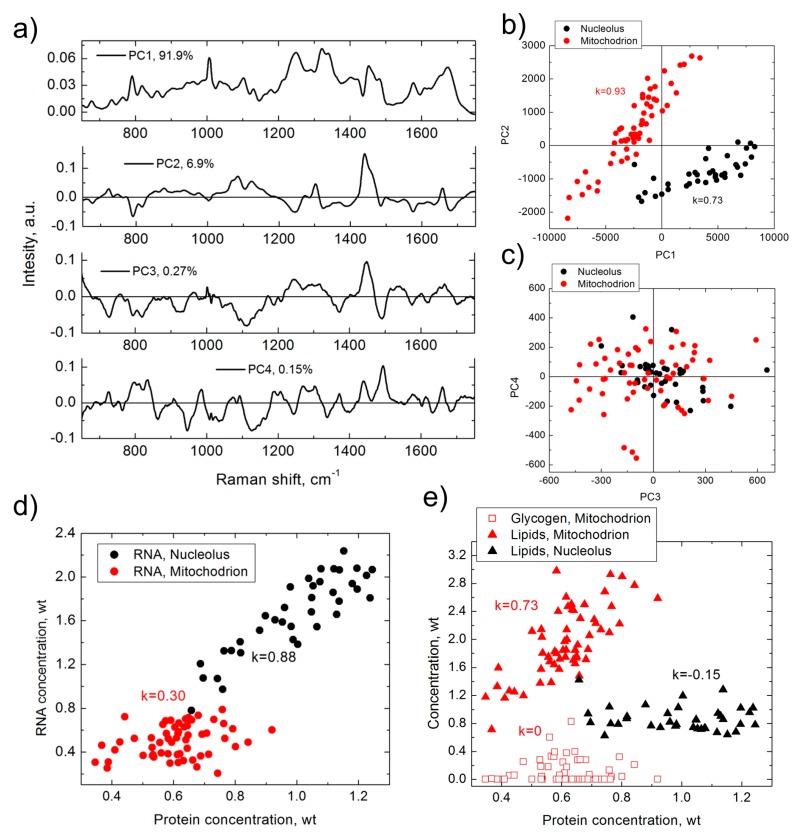
Results of PCA (**a**–**c**) and BCA (**d**,**e**), obtained from the same set of Raman spectra measured in nucleoli (black) and mitochondria (red) of HeLa cells. (**a**) first four principal components (corresponding contribution is shown in graph); (**b**) PC2 versus PC1 score distribution; (**c**) PC3 versus PC4 score distribution; (**d**) RNA versus proteins concentration; (**e**) lipid/glycogen concentrations versus protein concentration. *k* is Pearson correlation coefficient. Weight units correspond to 100 mg/mL for proteins and 20 mg/mL for RNA, lipids and glycogen.

The advantages of using BCA over any implicit analysis method can be demonstrated with the following example. The same Raman data measured in two different organelles, nucleoli and mitochondria, of HeLa cells were analyzed using the PCA and BCA techniques (Figure 1).

As demonstrated in Figure 1, although the first four principal components (Figure 1a) add up to 99.2% of the PCA model, they do not represent any specific biomolecular spectrum and resemble a combination of protein, DNA, RNA, and lipids spectra with either positive or negative signals. The PCA approach can identify a separation of PC2/PC1 clusters for nucleoli and mitochondria (Figure 1b), which demonstrates difference in spectral composition of these two organelles. In addition, the figure shows a strong correlation between PC1 and PC2 in nucleoli and mitochondria, but the biomolecular nature of this correlation is not clear. The PC3 versus PC4 score plot (Figure 1c) demonstrates almost isotropic 2D distributions.

In comparison, analysis of the same set of data with BCA delivers more abundant information of the biomolecular composition of the organelles. BCA was able to identify the local concentration of proteins, RNA, and lipids for each organelle, as well as of glycogen in mitochondria. The data also indicate a weak concentration of DNA in each organelle, although it is below the limit of detection threshold. BCA detected significant differences in the biomolecular makeup of nucleoli and mitochondria (Figure 1d,e). BCA further shows that RNA concentration in nucleolus is strongly correlated with that of proteins, while in mitochondrion this correlation is moderate. At the same time, a strong correlation is observed between the concentrations of lipids and the proteins in mitochondria, but not in nucleoli. In addition, there is no correlation between glycogen and proteins in mitochondria.

Based on this simple example, we can summarize that BCA definitely outranks PCA in its analysis capabilities. Although BCA has not been widely used by the biomedical scientific community, and, as follows from publications, is little known in comparison to PCA. The reason for this will be discussed in the next section.

## 3. BCA Basics

### 3.1. Challenges

Generally, the requirement to know all the chemical components of the cellular compartment for modeling a priori is challenging. The composition of intracellular environment is complex and variable, and it requires a significant effort to choose the proper set of component spectra to have a satisfactory model fit to the real spectrum. Depending on the accuracy of this component set, the proximity of fitting between the measured spectrum and the model spectrum will be close, but the model may not represent reality. In practice, model validation is necessary to establish how accurately the spectral fitting has been performed. Usually the residual profile between the model and the real spectrum can be used as a merit of the model quality. In this regard, to overcome the challenge of biochemical diversity in a live cell to properly model the complex Raman spectrum, the number of components can be limited to those which significantly contribute to the resulting Raman intensities. Evaluation of other minor contributors can be studied by analysis of the residual profile.

Besides assuming the number of Raman components used in modeling and the real number of biomolecules of a particular type at that particular location, another major source of the residual spectrum arises from the distinction between Raman biomolecular spectra in the sample and that in the corresponding model. Variations in the amino acids composition of proteins, nucleotide sequences in DNA and RNA, different lipids, small molecules and ions will all introduce variations to the measured Raman spectrum. The use of real Raman spectra of biomolecules as model components is an optimal choice for the modeling. However, in this case, the procedure of the extraction of full set of organellar molecular constituents is required, which is in practice extremely difficult. Isolation and purification of organellar proteins is a sophisticated procedure [41], accompanied by unavoidable losses, alterations and contamination of extract by constituents from other organelles [34]. A similar concern is valid for nucleic acids. Keeping in mind, that the closer the model is to real component spectrum, the better model fit is, a proper selection of the model components profiles is extremely important.

Considering the challenges discussed above, there is a common strategy to appropriately deconvolute a cellular Raman spectrum when using BCA—the establishment of biomolecular components and their Raman profiles, whose contribution in resulting Raman intensities is significant. To our knowledge, an implementation of this strategy has not been completed, which impedes the wide application of BCA for biomedical applications. Besides, there are two other important issues for satisfactory BCA employment. First, the removal of the uncontrollable background accompanying the weak Raman signal of bio-constituents, which can distort spectral shape and compromise further analysis. Second, the calibration procedure, which is required for absolute concentration probing.

Further, we will review and discuss the methods to overcome these issues, which are important steps for standardization of the BCA protocols, and are necessary to implement a BCA toolbox for Raman spectrometers.

### 3.2. Selection of Biomolecular Components

In practice, most publications on biomolecular modeling of intracellular Raman spectra are based on a set of the major classes of macromolecules—proteins, nucleic acids, lipids and saccharides, which collectively make major contribution to the non-elastic scattering intensities [42]; thereby, component set can be limited to five spectra of these biomolecules [33,34,35,36,37,38,39]. Tissue modeling, especially extracellular matrix, requires additional specific proteins (e.g., collagen, elastin, tropomyosin) lipids (e.g., cholesterols, triglycerides), carotenes and non-organic constituents [29,30,31,32,43,44].

### 3.3. Component Raman Spectra

Nucleic acids. Nucleic acids, in spite of difference in sequences and secondary structures, have similar Raman spectra [39]. In this regard, commercially available DNA (Calf thymus DNA, Salmon sperm DNA) and RNA (*S. cerevisiae* RNA, Calf liver RNA, and/or t-RNA) solutions can be used for the measurement of their respective Raman component profiles [33,34,39,40]. At the same time, DNA and RNA components can be measured in the solutions of nucleic acids extracted directly from the cells of the studied cell line [35].

Lipids. Combination of two compounds (triolein and phosphatidylcholine [38], mixture of phosphatidylcholine and cholesterol [37]), lipid extract from tissue liver cells [33], bovine heart lipid extract [39,40], or lipids isolated from the measured cells [34,35] have been used for the lipid component spectral model profile.

Saccharides. Raman spectra of glycogen, either commercial product [33,38] or extracted from cells [35], have been used as a model component.

Proteins. A number of known proteins and their mixtures were used as protein Raman spectral components. Specifically, actin and albumin [38]; actin, collagen, human serum albumin, chymotrypsin [37]; bovine serum albumin [40]; collagen, elastin, actin, myosin, tropomyosin [29]; proteins isolated from MR1 or Rat1 frozen cells [33], protein isolated from the fibroblast cells [34].

Thus, in practice the choice for Raman model components for nucleic acids, saccharides, and lipids is not troublesome and the spectrum can be measured by using commercially available compounds, or from ingredients, isolated from the cells by simple standard protocols. At the same time, selection of the protein component is relatively complex procedure. Diversity of cellular proteins results in multiple variations of the Raman profile depending on the type of cell or cellular organelle. For example, mixtures of several proteins were used for modeling with limited success. Even Raman spectrum of proteins, extracted from cells and used as a model component, do not guarantee precise matching between the model and measured Raman spectra [34].

Nevertheless, well-established component spectra for nucleic acids, lipids and glycogen allows for the assumption that after subtraction of contributions of all these weighted components from the measured Raman spectrum, the residual Raman profile can be assigned to the contribution of various proteins which comprise 70–80% of the dry weight of a cell [45], and that of other minor biomolecules. Therefore, this spectral profile may play a role of an ideal model component considering as “organellar proteins” [39,46].

**Figure 2:**
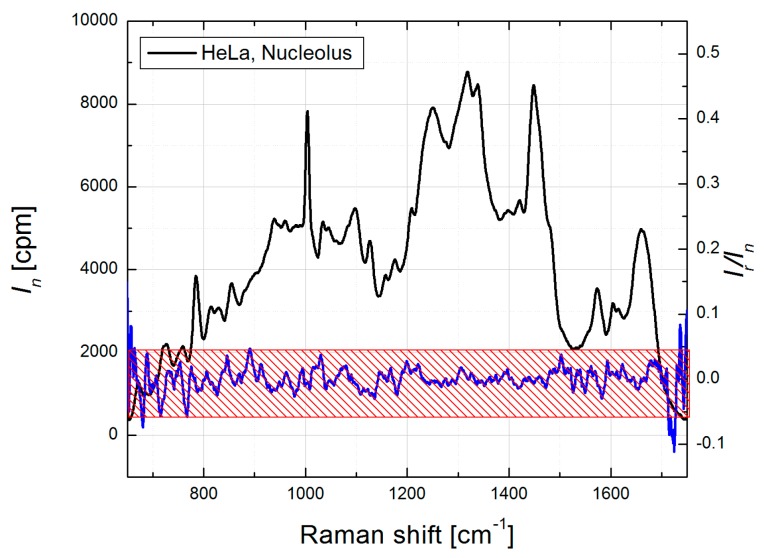
Mean Raman spectrum of HeLa nucleolus, collected from 8 single cells (black curve, left *Y*-axis) and ratio *I_n_/I_r_*, (blue curve, right *Y*-axis), where *I_n_* and *I_r_* are corresponding intensities of mean nucleolus and residual spectra. Red patterned box shows ±5% margins at the left *Y* axis.

To verify this approach for protein component “simulation”, we performed biomolecular modeling in HeLa cells [39,46] and selected Raman spectra profiles for five major types of biomolecular components. Then this set of biomolecular component spectra was used to model specific organelles of WI-38 cells, HCT116 WT and HCT116 Bax−/− colon cancer cells, dermal fibroblasts, induced pluripotent stem cells and embryonic stem cells [47,48,49]. In all of these studies where the BCA algorithm was applied, (i) the same Raman spectra profiles for RNA, DNA, lipids and glycogen components for all organelles; but (ii) organelle specific protein profiles, obtained as residual spectrum after subtraction of weighted spectra of other biomolecular components from organellar spectrum and averaged over multiple measurements, were used. The good quality of spectral modeling in different cell lines with the same component set supports a universal format of this selection. As an example, Raman spectrum of HeLa nucleolus and corresponding spectral ratio of Raman and residual curves are shown (Figure 2). The residual spectrum was obtained by subtraction of the model profile from the nucleolar Raman spectrum. For the modeling, Raman profiles of calf thymus DNA, RNA extracted from HeLa cells, bovine heart lipid extract, and nucleolar protein component, obtained as discussed above, were used as the component spectra of DNA, RNA, lipids and proteins, respectively. In Figure 2, the amplitudes of the ratio profile (*I_r_/I_n_*) are within 5%, except at the edges (<680 and >1700 cm^−1^), which is close to standard error for Raman measurements in a uniform protein sample ([39], Supplementary Information).

Therefore, to ensure the quality of fit for organellar BCA, we recommend that the residual intensities over the significant wavelength range should not exceed the standard error produced by Raman spectrometer in the measurements using a uniform protein mixture, since most of the subcellular Raman data is based on the protein content.

**Figure 3:**
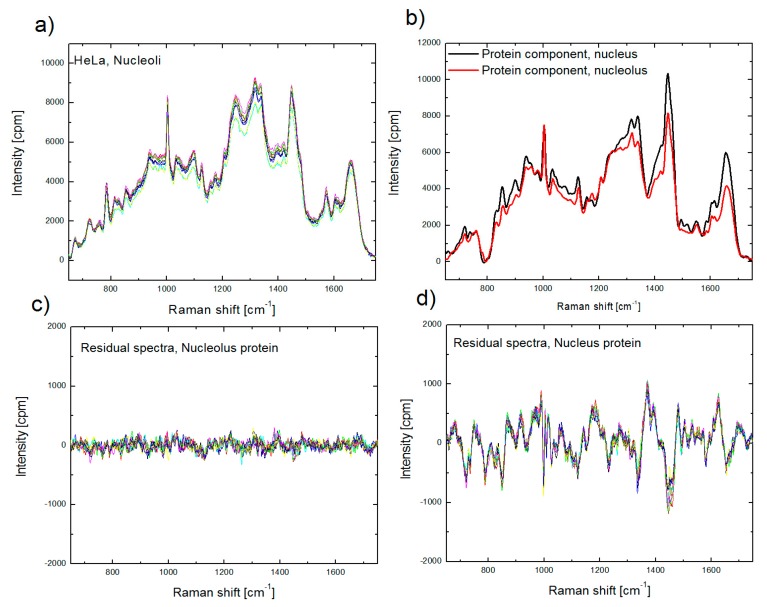
Raman spectra of nucleoli, collected from nine single cells (**a**); protein spectral component for nucleolus (red) and nucleus (black) used in BCA (**b**); residual spectra obtained after BCA of spectra, shown in (**a**), using proper (nucleolus) protein component (**c**) and improper (nucleus) protein component (**d**).

At the same time, using improper protein component in BCA, for example a nuclear (extra-nucleolar) protein component for biomolecular analysis of nucleolus, often results in larger intensity in the residual profile (Figure 3). This outcome suggests that the sensitivity of BCA is sufficient to recognize the differences between proteomes of cellular organelles [50], and enables preliminary organellar proteome analysis, which will be discussed in Section 4.

### 3.4. Calibration of Biomolecular Components

This step is important to analyze bio-constituents in absolute concentrations. All Raman micro-spectrometer systems differ from each other by photodetector sensitivity, dispersion of spectrometers, and optical losses in excitation and detection channels. This requires calibration of absolute biomolecular component concentrations for each device. Usually this procedure includes careful measurement of Raman spectra of biomolecular solutions with known concentrations. Then calculated from the calibration curve, which is linearly dependent on Raman spectra intensity (in CCD counts) on concentration, a biomolecular concentration value is assigned to each specific component spectrum. Calibration of micro-spectrometers is usually performed by measuring Raman spectra of the solutions of the commercially available biomolecular products with known concentration.

### 3.5. Background Subtraction

The sources of uncontrollable background generated during confocal Raman measurements of bio-constituents are: (i) Raman scattering from the substrate and the medium; (ii) light scattering, other than Raman; (iii) intrinsic fluorescence (autofluorescence) of biomolecules; and (iv) detector dark noise.

The most direct strategy to reduce the Raman spectra background is using a combination of: (i) appropriate laser excitation wavelength; and (ii) proper substrate material for cell growth [51]. Lasers that emit in the blue-green visible range excite higher autofluorescent signal, whereas lasers that emit in the infrared wavelength region produce larger background from the substrate material and optical elements within the excitation channel. For this reason, red lasers (630–680 nm) are optimal sources for Raman excitation, producing moderate background signal. Materials with low Raman scattering (CaF_2_, BaF_2_ or MgF_2_) are able to reduce the background signal, generated by substrate itself [29,37]. Unfortunately due to the high cost of slides produced from these materials, its application is limited. Therefore, growing cells on the luminescence free Raman-grade glasses most commonly used.

The post-processing algorithm to remove the background from the recorded spectra is generally accomplished by subtraction of preliminary measured spectrum of the medium (buffer) or empty sample cell, and spectrum of fluorescence, which can usually be modeled with a polynomial filter [29,33,34,35,36,37,38]. The contribution of the medium and the substrate to the measured background spectrum strongly depends on the proximity of the substrate to the probing point (i.e., the center of the laser beam waist formed by the microscope objective). For high aperture 100× microscope objectives with oil immersion, the critical distance between the probing point and the substrate is approximately 1 micron. If the distance is closer, the contribution of the glass substrate to the background could be significantly high, resulting in distortion of the Raman spectrum even after background subtraction. For distances larger than ~6 microns, the contribution of the substrate to the background spectrum is minor and the background is mostly represented by the Raman and Mie spectra of water and optical elements, which is less distortive to bio-constituents spectra.

All background components, accurately recorded, smoothed and normalized, can be added to the set of biomolecular spectral components (see Section 3.2) for the modeling of the measured spectrum using the chosen fitting algorithm.

### 3.6. Fitting Algorithm

A nonlinear least squares algorithm or its modification (Levenberg-Marquardt) is usually used to fit the model to the measured Raman spectra [29,33,34,35,36,37,38].

## 4. BCA Applications

### 4.1. Cell Analysis

Early BCA applications were mostly devoted to the classification of cancerous and noncancerous cells based on the differences of biomolecular content. Both PCA and BCA methods were utilized to analyze the Raman spectra measured from live, apoptotic, and necrotic leukemia cells [38]. Ong, et al. established the capability of BCA to detect fine biochemical changes in whole cell’s Raman spectra and obtain impressive accuracy in classification of cells. At the same time, the changes in the contribution of principle components in PCA were difficult to interpret. Reproducible differences in the biochemical composition that are not readily apparent by visual analysis of vibrational bands in the spectra were found for both tumorigenic and non-tumorigenic rat fibroblast cell lines [33,34,35]. The differences included an increased fraction of protein and nucleic acids in the tumorigenic cells, with a corresponding decrease in lipid and glycogen fractions. 

In addition, BCA has been used to estimate biochemical changes due to necrosis in MEL-28 human melanoma cells growing in vitro [36]. A decrease in the relative amount of lipid and RNA, and an increase in the relative protein content, were observed in dead cells. A comparison of the spectra indicated the existence of conformational changes in protein and nucleic acids in dead cells.

One important application of BCA is the ability to verify the suitability of immortal cultured cell lines as models for primary cells. The use of primary cells can be hampered by an unreliable supply; the difficulty of performing isolation and culture procedures in vitro; limited division number and loss of phenotype with increasing time in culture. To overcome these limitations, BCA was used to characterize live A549 cells as a model for primary human pulmonary alveolar type II epithelial cells (ATII), and a transduced type I (TT1) cell line as a model for alveolar type I (ATI) cells [37]. The results of the BCA modeling suggest that A549 cells are not a good model for ATII cells, but TT1 cells do provide a reasonable model for ATI cells.

### 4.2. Tissue Analysis

Biochemical assessment of cellular and extracellular morphologic structures in situ is one of the efficient methods to discriminate between normal and abnormal tissue sites. BCA was employed to obtain quantitative chemical information on coronary arteries and to develop diagnostic algorithms that accurately discriminate between nonatherosclerotic tissue, noncalcified plaques, and calcified plaques on the basis of relative weights of calcium salts and total cholesterol [43]. This method provided reliable histochemical information about peripheral and coronary arteries, which may help to identify rupture-prone plaques before the onset of symptoms and allow aggressive and directed intervention [44]. Based on results of BCA of human coronary atherosclerosis, a conclusion was made that modeling may be used to analyze the morphologic composition of atherosclerotic coronary artery lesions [29].

Raman microspectroscopic modeling of human breast tissue was employed for breast cancer diagnosis in vivo [30]. Lipid quantification in prostate cancer tissue by BCA, revealed a correlation between lipid accumulation and tumor staging, which provided a quantitative marker for prostate cancer diagnosis [52].

### 4.3. Analysis of Organelles

High 3D resolution of confocal Raman microspectroscopy makes it an appropriate analytical tool for local molecular composition, suitable for quantitative biomolecular analysis and real-time biomolecular monitoring in distinct subcellular structures such as cytoplasmic organelles and structure–function compartments of the cell nucleus.

Confocal Raman microscopy does not always ensure accurate probing of subcellular structures due to difference between the size and shape of organelles and the probing dimensions. Besides, several large organelles including nucleoli, mitochondria, Golgi complex, and endoplasmic reticulum, are non-uniform, and some variation in the Raman spectra can be detected in the different parts of the same organelle. Nevertheless, the spectra obtained in the same type of organelle demonstrate a significant similarity, which may be associated with specific molecular microenvironment in these subcellular structures.

**Figure 4:**
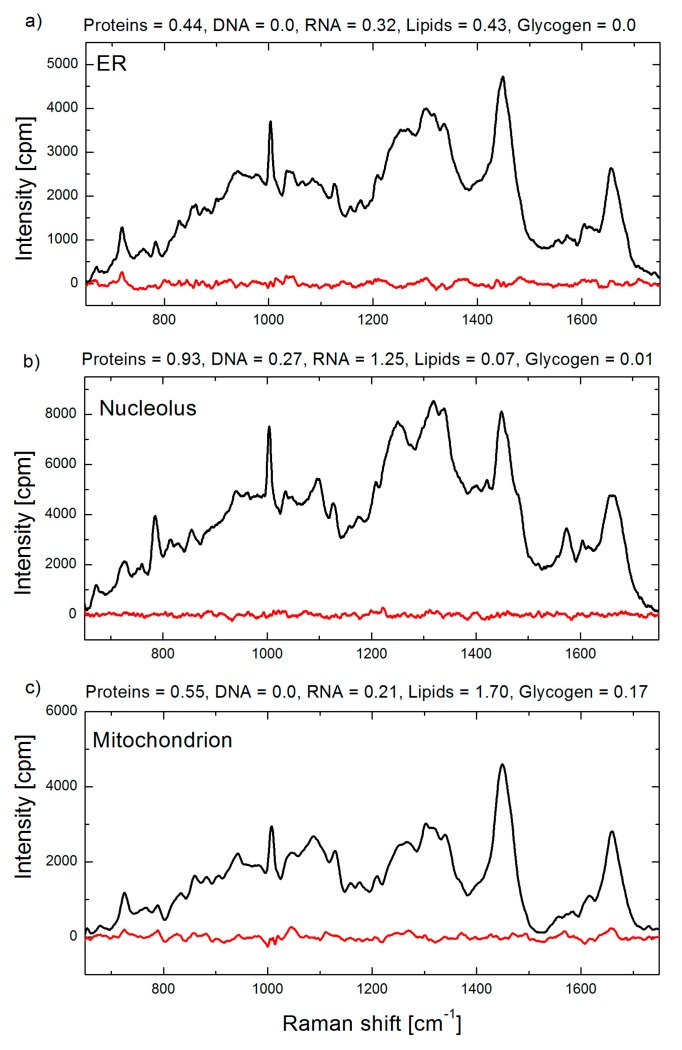
Raman spectra of a single organelle: (**a**) endoplasmic reticulum; (**b**) nucleolus; and (**c**) mitochondrion in live HeLa cells. Raman spectra are shown in black and corresponding residual spectra obtained by BCA procedure are shown in red. Biomolecular weights obtained by BCA are indicated in arbitrary units, which for this example correspond to 100 mg/mL for proteins, and 20 mg/mL for DNA, RNA, lipids and saccharides, respectively.

The first application of BCA in organellar analysis, to the best of our knowledge, was performed by our group at the Institute of Lasers, Photonics and Biophotonics (ILPB), the University at Buffalo. In the most of our studies we used our BCA toolbox coded in Matlab software. Raman spectral concentration calibration was performed using bovine serum albumin, calf thymus DNA, S. cerevisiae RNA (Sigma Aldrich, St. Louis, MO, USA), and bovine heart lipids (Avanti Polar Lipids, Alabaster, AL, USA), with unit weight of 100 mg/mL for proteins and 20 mg/mL for RNA, DNA and lipids concentrations. The BCA toolbox yields a set of biomolecular weights (e.g., proteins, DNA, RNA, lipids and glycogen) and residual profile for each analyzed spectrum. Representative BCA processed spectra and output data are shown in Figure 4.

We used this method to study the macromolecular organization of the cell nucleus throughout the cell cycle in HeLa cells. In this study, site-specific concentrations of proteins, DNA, RNA, and lipids were estimated in nucleoli, nucleoplasmic transcription sites, nuclear speckles, constitutive heterochromatin domains, mitotic chromosomes, and extrachromosomal regions of mitotic cells [53]. We were surprised to find that the local concentration of proteins did not increase during DNA compaction and that postmitotic DNA decondensation is a gradual process, continuing for several hours.

As a continuation of our site-specific probing strategy, we focused on the characterization of complex molecular organization of the nucleolus, which is the largest structure–function compartment of the cell nucleus [39]. BCA was used to determine the contribution of each major type of macromolecule. A notable cell-to-cell variability in the macromolecular composition of nucleolus was found. At the same time, a correlation between the concentrations of major types of biomolecules in this nuclear compartment was observed. In particular, the average concentration of RNA increases along with an increase in the protein concentration, while an inverse dependence exists between the concentrations of RNA and DNA. In another study, we investigated the effect of cell fixation on the macromolecular profiles of nucleoli [46]. The fixation of biological samples is an essential technique necessary to “freeze” in time the intracellular molecular content. An accurate, sensitive, and comprehensive characterization of changes in biomolecular composition, occurring during fixation, is crucial for proper analysis of experimental data. To understand what kind of changes may be induced in the nucleolar biomolecular profile during cell fixation, we applied BCA for Raman spectral measured in the same nucleoli of HeLa cells before and after fixation by either formaldehyde solution or by chilled ethanol [46]. Fixation with formaldehyde did not strongly affect the Raman spectra of nucleolar biomolecular components, but significantly decreases the nucleolar RNA concentration. At the same time, ethanol fixation leads to changes in the secondary structure of nucleolar proteins and to concentration increase of proteins and RNA.

Next, we have demonstrated that microRaman-BCA enabled real-time monitoring of RNA synthesis in the nucleoli of live cultured cells [54]. In this study, we hypothesized that the cell to cell variations in concentration of nucleolar proteins and RNA are connected with RNA synthesis level in this compartment. We tested our hypothesis by monitoring the biomolecular composition in a single nucleolus. Using the microRaman-BCA technique, there were found high and rapid fluctuations of ribosomal RNA (rRNA) production rates. The changes in the rRNA output were synchronous for ribosomal genes located in separate nucleoli of the same cell. Numerical modeling demonstrated that the production of rRNA and ribosomal proteins can be coordinated, regardless of the fluctuations in rRNA synthesis. Our quantitative data revealed a unique interplay between the inherently stochastic rates of RNA synthesis and coordination of gene expression.

The nucleolar molecular signature was also compared between primary human skin fibroblasts, induced pluripotent stem cells (iPSCs) derived by reprograming of skin fibroblasts, and human embryonic stem cells [48]. In this research, we reported that (i) cultured fibroblasts obtained from different human subjects, share comparable concentrations of proteins, RNA, DNA, and lipids in the molecular composition of nucleoli; and (ii) the nucleolar molecular environment is changed during reprograming of fibroblasts to iPSC. The transition from skin fibroblasts to iPSCs is accompanied by a statistically significant increase in protein concentrations (~1.3-fold), RNA concentrations (~1.3-fold), and DNA concentration (~1.4-fold). We concluded that the nucleolar molecular content is correlated with the cellular differentiation status.

Subsequently, we chose to study the transformations of the macromolecular landscape in the mitochondria during DNA-damage-induced apoptotic cell death [47]. During apoptosis, significant changes occur in the concentrations of RNA, DNA, protein, and lipid constituents of mitochondria. These changes include replication of mitochondrial DNA, as well as production of cellular reactive oxygen species (ROS). Moreover, the upregulation of polymerase-γ, mitochondrial helicase Twinkle, and mitochondrial transcription factor A in response to drug-induced DNA damage, correlated with increased mitochondrial RNA synthesis, thus demonstrating a previously unknown dynamic correlation between macromolecular structure of mitochondria and progression of apoptosis.

### 4.4. Towards Non-Invasive Proteomics In-Situ

As we discussed above, the ‘cellular proteins’ component corresponds to the remaining spectrum obtained after the subtraction of the model weighted spectra of DNA, RNA, lipids (and saccharides in cytoplasm) from the measured spectra of the organelle. Usually this Raman profile contains integral Raman contributions from (i) a variety of different type of proteins; (ii) minor bio- and other molecular components; and (iii) spectral deviations of model components (DNA, RNA, lipids and saccharides) from real biomolecular components. In the last case, the accuracy of the analysis depends on the choice of component spectral profiles, and the presence of misfit can be manifested in systematically appearing bands in the residual spectra, co-located with major spectral bands of these biomolecular components. This shows how component spectrum correction can increase the accuracy of the model fit.

Minor molecular components, presenting at negligible concentrations, make insignificant contribution to the integral Raman signal, just increasing background. However, abundant cellular proteins make overwhelming contribution to the “proteins” model component. A comparison of the normalized protein component, obtained in different organelles, or for the same organelle in different cells, allows for the analysis of specific differences other than the deviations of biomolecular concentrations. For example, a comparative analysis of Raman profiles of HeLa nucleolar protein component and BSA [39] showed significant differences beyond the standard experimental deviations. The differences are evident in the Raman bands assigned to the aliphatic side chains and β-sheet conformation, where the intensity is higher in HeLa nucleolar protein spectrum, and Amide III assigned to α-helix conformation, where the intensity is substantially higher in the BSA spectrum.

Systematic studies of protein components in three major cellular organelles—nucleoli, endoplasmic reticulum and mitochondria, reveal substantial spectral differences of organellar proteomes, while protein spectra of the same type organelle in different cell lines demonstrated less obvious differences [50].

The BCA technique allows for uncovering of changes of protein component occurring in the course of severe cellular alterations induced by external impact or internal processes. For example, ethanol fixation triggers a decrease in the α-helical structure of nucleolar proteins and an increase in the β-sheet conformation fraction of the proteins [46]. Changes of nucleolar proteins are also found during cell reprogramming [48]. In iPSCs, we observed a higher signal from tryptophan, with an increase in the random coil and α-helix protein conformations. At the same time, nucleolar protein conformations observed in human embryonic stem cells and iPSCs are similar.

The structural analysis of proteins of mitochondria during apoptosis [47] demonstrated a decrease in α-helix content, and an increase in the levels of random coils and β-sheets conformations.

## 5. BCA Perspective

A shift to the individual organelles as a target for Raman microspectrometry, brings a new dimension of this technique for bio-applications. Being oriented as a specific-organelle tool, microRaman-BCA can be classified as an omic technology, aimed at collectively characterizing and quantifying pools of organellar biological molecules and, thus, provides information on cellular processes including metabolism, cell response to external factors and drug-cell interactions. At the same time, this method is relatively underrepresented in research labs due to the reasons discussed in Section 3, most notably due to the absence of (i) a reliable and universal biomolecular component set; (ii) protocol of measurements, required for high quality BCA; and (iii) algorithms for automated spectral processing.

In our opinion, it is quite feasible to establish the set of universal biomolecular component profiles, which can be used for general BCA of the organelles suitable for confocal Raman measurements, and to develop a user friendly interface of BCA software together with visual control of modeling quality.

As the first step for implementation of the BCA unit in commercial micro-Raman systems, we developed a semi-automated BCA toolbox for a microRaman spectrometer to generate biomolecular datasets of subcellular sites of mammalian cells in real time, which can be used in further statistical and correlation computations. The software toolbox was coded in Matlab and has an input dataset, which includes Raman components for three cellular organelles (nucleolus, mitochondrion and endoplasmic reticulum). We verified the capabilities of microRaman-BCA toolbox for molecular profiling at a single organelle level in normal and cancer cell lines [49]. Preliminary results of this project showed promising perspectives for the developed toolbox. We found, that the macromolecular profiles of cellular organelles can be used as a set of quantitative markers for assessment of cellular physiologic state and identification or characterization of cellular heterogeneity, a fundamental property of cellular populations. In the next step of this project, efficiency of microRaman-BCA should be validated for analysis of biochemical variations between cells obtained from different origins, including cancer cells.

The key goal of further research is to establish the BCA-based Ramanomics as an independent omics discipline, harnessing its unique capabilities for non-invasive analysis of subcellular structures, by integrating it together with conventional bioanalytical techniques. Collaboration between academic research groups and manufacturers of Raman hardware would make a significant contribution to achieve this goal.
